# Non-active antibiotic and bacteriophage synergism to successfully treat recurrent urinary tract infection caused by extensively drug-resistant *Klebsiella pneumoniae*

**DOI:** 10.1080/22221751.2020.1747950

**Published:** 2020-04-02

**Authors:** Juan Bao, Nannan Wu, Yigang Zeng, Liguang Chen, Linlin Li, Lan Yang, Yiyuan Zhang, Mingquan Guo, Lisha Li, Jie Li, Demeng Tan, Mengjun Cheng, Jingmin Gu, Jinghong Qin, Jiazheng Liu, Shiru Li, Guangqiang Pan, Xin Jin, Bangxin Yao, Xiaokui Guo, Tongyu Zhu, Shuai Le

**Affiliations:** a Shanghai Institute of Phage, Shanghai Public Health Clinical Center, Fudan University, Shanghai, People’s Republic of China; b Department of Microbiology and Immunology, Institutes of Medical Sciences, Shanghai Jiao Tong University School of Medicine, Shanghai, People’s Republic of China; c Department of Microbiology, Army Medical University, Chongqing, People’s Republic of China; d Department of Pathology, The Second Affiliated Hospital of Army Medical University, Chongqing, People’s Republic of China; e Department of Pharmacy, Zhongshan Hospital, Fudan University, Shanghai, People’s Republic of China; f Shanghai Key Laboratory of Organ Transplantation, Zhongshan Hospital, Fudan University, Shanghai, People’s Republic of China

**Keywords:** Bacteriophage, phage therapy, antibiotic resistance, urinary tract infection

## Abstract

We report a case of a 63-year-old female patient who developed a recurrent urinary tract infection (UTI) with extensively drug-resistant *Klebsiella pneumoniae* (ERKp). In the initial two rounds of phage therapy, phage resistant mutants developed within days. Although ERKp strains were completely resistant to sulfamethoxazole-trimethoprim, the combination of sulfamethoxazole-trimethoprim with the phage cocktail inhibited the emergence of phage resistant mutant *in vitro*, and the UTI of patient was successfully cured by this combination. Thus, we propose that non-active antibiotic and bacteriophage synergism (NABS) might be an alternative strategy in personalized phage therapy.

Antibiotic resistant bacteria pose a major challenge in clinics. Urinary tract infection (UTI) with extensively drug-resistant *Klebsiella pneumonia* (ERKp) is a challenging infectious complication in immunocompromised patients, such as transplant recipients, and patients with cancer and diabetes [1–3]. The efficacy of antibiotics in treating UTIs is decreasing.

Lytic phage is a promising alternative approach in treating bacterial infections [4,5] and has been applied in personalized phage therapies and a randomized double-blind phage therapy clinical trial [6,7]. However, phage therapy still has limitations. The development of phage resistance is one challenge [8,9]. Thus, novel phage therapy strategies are needed to overcome phage resistance of extensively drug-resistant bacteria.

In May 2019, a 63-year-old female with recurrent UTI, was transferred from a local hospital in Shenzhen to Shanghai Public Health Clinical Center for phage therapy. The pathogen of UTI was determined as ERKp, which resisted all tested antibiotics, except tigecycline and polymyxin B. The medical history of the patient was described with a long history of type 2 diabetes and hypertension. The initial occurrence of UTI emerged in October 2018, which led to acute pyelonephritis and infectious shock. After critical care treatments, including tigecycline administration, the UTI was not cured and became persistent. It was treated with antibiotics using tigecycline, levofloxacin, and sulfamethoxazole. Moreover, complications due to antibiotic consumption have become increasingly severe. Thus, the patient was enrolled in phage therapy clinical trial after involvement evaluation and informed consent.

We isolated an ERKp strain (CX7224) from the urine of the patient. The antibiotic susceptibility pattern of this isolate was identical to the previous isolates from the patient (Table 1), and the sequence type (ST) was classified as ST11. We performed phage spot test using double-layer agar method to screen candidate phages from our collection of over 200 lytic phages against *K. pneumoniae*. We used five lytic phages, namely, SZ-1, SZ-2, SZ-3, SZ-6, and SZ-8 (Table 1), which have efficient lytic activities against the CX7224 strain, as cocktail I for the first round of phage therapy. We made a regimen of antibiotic withdrawal and 5 days phage therapy from 9–13 May 2019. On a daily basis, we administered 50 millilitre (mL) cocktail I with equally mixed phages (5 × 10^8^ plaque forming units per mL of each phage) by bladder irrigation.

**Table 1.** Bacteriophage and antimicrobial susceptibility of representative clinical isolates.

	Strains	CX7224 (May 5)	CX8070 (May 14)	CX10301 (June 30)
Bacteriophage sensitivity in clinical isolates^a^	vB_KpnS-SZ-1	+	−	+
	vB_KpnS-SZ-2	+	−	+
	vB_KpnM-SZ-3	+	−	+
	vB_KpnS-SZ-6	+	−	+
	vB_KpnP-SZ-8	+	−	+
	vB_KpnM-Kp165 vB_KpnP-Kp166 vB_KpnP-Kp167 vB_KpnM-Kp168 vB_KpnM-Kp169	+ + + + +	+ + + + +	− − − − −
	vB_KpnM-Kp152 vB_KpnP-Kp154 vB_KpnP-Kp155 vB_KpnP-Kp164 vB_KpnS-Kp6377 vB_KpnM-HD001	+ + + + + +	+ + + + + +	+ + + + + +
	**Antibiotic**^b^			
Antimicrobial susceptibility of clinical Isolates (MIC [µg/mL])^c^	TGC PMB PIP-TAZ	S (0.5) S (0.25) R (≥128)	S (1) S (0.5) R (≥128)	S (1) S (0.5) R (≥128)
	TOB	R (≥16)	R (≥16)	R (≥16)
	SMZ-TMP	R (≥320)	R (≥320)	R (≥320)
	AZT	R (≥64)	R (≥64)	R (≥64)
	CTX	R (≥64)	R (≥64)	R (≥64)
	GEN	R (≥16)	R (≥16)	R (≥16)
	CAZ,	R (≥64)	R (≥64)	R (≥64)
	LEV	R (≥8)	R (≥8)	R (≥8)
	AMI	R (≥64)	R (≥64)	R (≥64)
	CIP	R (≥4)	R (≥4)	R (≥4)
	IPM	R (≥16)	R (≥16)	R (≥8)

^a^Phage sensitivity based on the double-layer agar plate assay. (+): phage forms clear plaques on double-layer agar; (−): phage does not form any plaque or form blurred plaques on double-layer agar.

^b^TGC, tigecycline; PMB, polymyxin B; PIP-TAZ, piperacillin-tazobactam; TOB, tobramycin; SMZ-TMP, trimethoprim-sulfamethoxazole; AZT, aztreona; CTX, ceftriaxone; GEN, gentamicin; CAZ, ceftazidime; LEV, levofloxacin; AMI, amikacin; CIP, ciprofloxacin; IPM, imipenem.

^c^R, resistant; S, susceptible.

Despite the apparent relief of the patient’s symptoms during the first round of phage therapy, ERKp strains were still detected at a very low level during the phage therapy. The ERKp strain CX8070 (ST11) isolated after phage therapy exhibited the same antibiotic susceptibility as CX7224 but is completely resistant to all the five phages of cocktail I (Table 1). This result indicated the potential selection stress of phages on the ERKps of the patient.

Cocktail II, which consisted of phages Kp165, Kp166, Kp167, Kp158, and Kp169, were prepared with full lytic coverage to the already isolated ERKp strain CX8070 from the patient. However, new ERKp isolates reemerged during the second round of phage therapy from June 3 to 7, 2019. These new isolates presented resistance to cocktail II, whereas the remaining isolates were resistant to all tested antibiotics, except tigecycline and polymyxin B. ERKp strain CX10301 (ST11) was completely resistant to all phages of cocktail II, and it was used for phage selection in the third round of phage therapy (Table 1).

The repeated reemergence of phage-resistant ERKps indicated that phage resistance was a serious issue in this patient and novel therapeutic strategies are needed. We speculated that screening some non- active antibiotics might enhance the treatment effect of phages. A new cocktail III with six lytic phages (Kp152, Kp154, Kp155, Kp164, Kp6377, and HD001) was selected for its full coverage of the ERKp strains. Several antibiotics frequently used in UTI treatment were tested to enhance phage therapy efficacy. Among these antibiotics, we identified trimethoprim-sulfamethoxazole (SMZ-TMP) as the one with a strong synergistic effect with cocktail III.

As shown in Figure 1(A), ERKp strain CX10301 growth was efficiently blocked by cocktail III until 12 h. However, the resistant bacteria became dominant and were nearly replenished at 24 h post-incubation. Growth curve of CX10301 exhibited resistance to SMZ-TMP alone even at high doses. Interestingly, combining cocktail III with moderate or high dose of SMZ-TMP (150 µg/mL SMZ, 50 µg/mL TMP or 300 µg/mL SMZ, and 100 µg/mL TMP) completely suppressed the growth of CX10301 for more than 24 h (Figure 1(B)).

**Figure 1.**
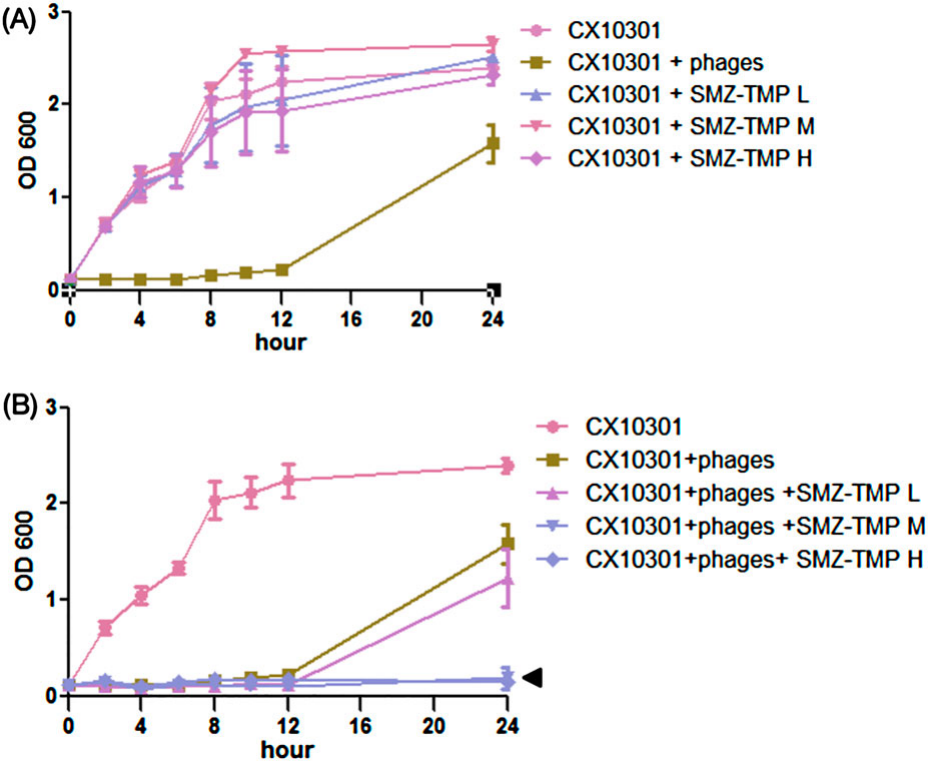
Growth curve of ERKp strain CX10301 under various treatments. **(A)** Six phages (Kp152, Kp154, Kp155, Kp164, Kp6377, and HD001, 5 × 10^8^ pfu/mL for each phage) were equally mixed to make a phage cocktail III. 10 mL of bacterial culture (OD600 = 0.1) was mixed with 100 µL of phage cocktail III. Cocktail III inhibits the growth of CX10301 for 12 h, and the resistant mutants developed to a high density within 24 h. Trimethoprim-sulfamethoxazole (SMZ-TMP) cannot inhibit the growth of CX10301 at three concentrations. (H = 300 µg/mL SMZ, 100 µg/mL TMP; M = 150 µg/mL SMZ, 50 µg/mL TMP; L = 75 µg/mL SMZ, 25 µg/mL TMP). **(B)** The combination of higher concentrations of SMZ-TMP (M and H) and cocktail III could significantly inhibit the emergence of phage-resistant mutants. The *in vitro* experiments were performed in Luria-Bertani liquid medium, and each experiment was repeated three times.

SMZ-TMP is usually applied in treating UTIs, and SMZ-TMP could reach an extremely high concentration in the urine than in the blood [10]. Considering these data, we developed a combined regimen of orally administrated SMZ-TMP (800 mg–160 mg) twice a day and bladder-irrigated cocktail III once a day for 5 days from July 2 to 6.

Finally, after this round of phage therapy combined with non-active antibiotics treatment, the pathogenic ERKp of the patient was completely eliminated and the recurrent UTI symptoms subsequently disappeared. No adverse event occurred during the whole period of phage therapy, and the patient was finally discharged at the end of the month. No signs of recurrence were observed for this patient under antibiotic-free conditions during 6 months of follow-up.

Phage therapy is an attractive approach for treating multidrug-resistant organisms. However, phage resistance is an alarming issue in phage therapy [9]. Phage resistance is quite common *in vitro* [11] and was observed very quickly in this patient. We infer that the quick development of phage-resistant mutants is likely due to the poor immunity of the patient. The synergy between the immune system and the phages is essential for the clearance of bacterial infection [12]. If the immunity is strong, the remaining minor phage-resistant mutants will be cleared. However, the number of immunocompromised patients is growing due to the growing number of transplant recipients, and patients with cancer and diabetes. Thus, phage resistance would be a common issue of phage therapy in the near future.

Here, we propose several novel phage therapy options for treating UTIs, especially when neither antibiotic nor bacteriophage cocktail could cure the UTIs.

If the ERKp is sensitive to some antibiotics that do not cause complications, we highly recommend combining the active antibiotic with phage cocktail in the treatment to minimize the possibility of regrowth of phage-resistant mutants. This issue has been discussed previously [13].

If no active antibiotic is available, we could test whether SMZ-TMP and phage cocktail could inhibit the emergence of phage-resistant mutants *in vitro*. Also, the mechanism by which high concentrations of SMZ-TMP inhibit phage resistance is worth investigating.

Another approach is to study the molecular mechanisms of phage-resistant mutants and select the appropriate phages against the mutants. Then, these phages are combined into a cocktail to constrain the appearance of the phage-resistant mutants [14]. Otherwise, we can select different phages that use different receptors to minimize the emergence of resistance, because the frequency to mutate multiple sites simultaneously is much lower and could potentially inhibit the emergence of phage resistance. Phage resistance of *K. pneumonia in vitro* was evolved through cell surface structure modification to block phage adsorption [15]. However, how does *K. pneumonia* resist phage infection *in vivo* is an interesting question, and we are investigating phage resistance mechanisms of CX8070 and CX10301, which were selected *in vivo*. In this case, we select phages based on the formation of a clear plaque via a phage spot test using a double-layer agar method, and the lytic activity was further validated by inhibiting bacteria growth in liquid culture. However, the molecular mechanisms are quite important for the rational design of a phage cocktail in the near future.

Overall, this case demonstrates the importance of bacteriophage resistance in phage therapy and suggests that the combination of SMZ-TMP and bacteriophage could be used to treat recurrent UTI. Thus, we propose non-active antibiotic and bacteriophage synergism (NABS) as a useful alternative strategy in personalized phage therapy. However, more questions in molecular mechanisms involved in phage therapy remains. Phage therapy with a larger sample of participants is warranted to support these therapeutic strategies.
